# Young Children Learning from Touch Screens: Taking a Wider View

**DOI:** 10.3389/fpsyg.2016.01078

**Published:** 2016-07-18

**Authors:** Silvia B. Lovato, Sandra R. Waxman

**Affiliations:** ^1^Center on Media and Human Development, Northwestern University, Evanston, ILUSA; ^2^Department of Psychology, Northwestern University, Evanston, ILUSA

**Keywords:** touch screens, very young children

## Abstract

Touch screen devices such as smartphones and tablets are now ubiquitous in the lives of American children. These devices permit very young children to engage interactively in an intuitive fashion with actions as simple as touching, swiping and pinching. Yet, we know little about the role these devices play in very young children’s lives or their impact on early learning and development. Here we focus on two areas in which existing research sheds some light on these issues with children under 3 years of age. The first measures *transfer of learning*, or how well children use information learned from screens to reason about events off-screen, using object retrieval and word learning tasks. The second measures the impact of interactive screens on parent-child interactions and story comprehension during reading time. More research is required to clarify the pedagogical potential and pitfalls of touch screens for infants and very young children, especially research focused on capabilities unique to touch screens and on the social and cultural contexts in which young children use them.

Scene from the New York City subway, 2015: A sweet mom is riding with her 3- or 4-year-old daughter. The girl asks, “Where are the stairs? When are we going up the stairs?” Instead of following the child’s lead (e.g., telling her about the stairs, looking for stairs together, explaining that stairs are at the ends of the stations because there are no stairs in the tunnels, etc.), the mom starts drilling her on the sounds of letters. “What word starts with A, what word starts with B”, etc. They got to O - a hard one - and the child got frustrated. At this point, the mom handed her a tablet with a video game, and she turned to her own phone.

Episodes like this one, shared by a colleague, have become increasingly common. After all, touch screens are everywhere, and even the most devoted parent sometimes needs to turn her attention elsewhere, even if only briefly. Moreover, we know that even before their first birthdays, infants *can* learn from material presented on screens, as witnessed by their success in lab-based tasks as diverse as perceptual discrimination, pattern detection and word learning.

Observations like these – in subways and in infant labs – raise fundamental questions for the 21st century. What exactly can infants and very young children learn from the screen? On the one hand, although several ‘educational’ programs for infants and young children claim to teach a variety of skills, evidence-based investigations reveal that most fall far short of their mark (Zimmerman et al., 2007; Robb et al., 2009; DeLoache et al., 2010; Neuman et al., 2014). On the other hand, a review of the infancy literature reveals that infants can indeed learn a great deal from screens, including new words for objects and actions (Barr et al., 2007; Yuan and Fisher, 2009; Arunachalam and Waxman, 2010). Perhaps even more remarkable, infants as young as 18 months of age – most of whom speak only in single word utterances – can use the (few) words they do know to learn new words, even when the entire task takes place on a screen (Ferguson et al., 2014).

Our goal here is to summarize what we know about the conditions under which infants and toddlers learn from interactions with touch screens. In contrast to the growing body of research addressing this issue in preschool-aged children (see Hirsh-Pasek et al., 2015 for a review), the evidence from very young children – especially those younger than three – remains sparse. Therefore, our goal is to review two research arenas in which considerable headway with this age-group has been made, and to highlight directions for additional research with infants and children under 3 years of age.

## Young Children’s Access to Touch Screens

Young children’s access to touch screens has increased rapidly and dramatically. In October 2015, the Pew Research Center reported that at least 83% of all 18- to 49-year-olds in the US – the age group most likely to be parents of young children – owned smartphones (Anderson, 2015). Another recent investigation focusing directly on low-income minority families from suburban Philadelphia with children ranging from 6 months to 4 years painted the same picture (Kabali et al., 2015): 83% of these families had tablets at home, 77% had smartphones, and 96.6% of the children had used these devices, many before their first birthdays. Two years earlier, the nationally representative Common Sense Media survey reported that 38% of children under 2 had used a mobile device (Rideout and Saphir, 2013). Clearly, touch screen devices are rapidly gaining a place in the lives of US families with young children.

Why the explosion now? For decades, attractive, interactive graphic interfaces have been available on home computers. But young children’s access to these was limited by both their cost [with the cost of hardware, software, and home internet contributing to the “digital divide” (Norris, 2001)] and by the fine motor skills and eye-hand coordination required to manipulate a keyboard and mouse. With the advent of touch screens on less expensive devices – smartphones and tablets – these financial and developmental barriers have been reduced: By their first birthdays, most children can become adept at touching, swiping and pinching on the screen. As a result, children’s access to touch screens has outpaced what we know about its effects – for better or worse – on early development.

### The Gap Between Children’s Touch Screen Use and What We Know about Its Developmental Consequences

Because the research on touch screen use has not kept pace with their steep rate of adoption, there is a gap in our knowledge of their developmental impact, especially in children younger than three.

Several companies have claimed that infants learn from using their devices or apps. Academic researchers, on the other hand, have been more skeptical, asking about what conditions are required to support infants’ and young children’s learning from screens (c.f., Richert et al., 2010; Hirsh-Pasek et al., 2015).

Recent evidence points to both the promise (e.g., Rosin, 2013) and challenges of touch screen use (e.g., Glaser, 2014; Honan, 2014). The American Academy of Pediatrics (2011) has continued to recommend that screen time be minimized for children younger than 2 years of age. Researchers from early childhood education, developmental psychology and the learning sciences have raised questions about the impact of touch screens on cognitive and social development. Other questions concern what children can (or cannot) learn from screen-based interactions.

There is no doubt that, for the most part, young children learn best from exchanges with caring adults. There is also growing evidence that children learn more from media when their caregivers are actively engaged in what is known as *joint media engagement* (Takeuchi and Stevens, 2011). Moreover, when devices, apps, and toys are noisy, they interfere with the kinds of interactions that are best-suited for language and cognitive development (Kirkorian et al., 2009; Zosh et al., 2015). Thus, when parents or caregivers are available, very young children learn best interacting with them, without the interference of noisy devices.

But how often are young children engaged with parents or caregivers while using touch screens? And what do children learn from touch screens when they use them alone, at times when parents strive to keep them occupied, amused or momentarily distracted from a source of conflict?

A review of the research with children younger than three reveals two distinct, but relatively comprehensive, lines of work. The first measures *transfer of learning*, or how well children use information learned on-screen to reason about events off-screen. The second measures learning from interactive screens on during reading time.

## Learning From Screens: The Power of Interaction

### Transfer Tasks

The now-classic transfer task, pioneered by DeLoache (1987, 1989, 1995) and DeLoache et al. (1997) was designed to measure young children’s ability to transfer information gleaned from one medium (e.g., a 3D model, picture, screen-based depiction) to the ‘real world’. In the classic model room task, children first played with an experimenter in a room. Next, they accompanied the experimenter to a different location (e.g., a room with a 3D model of the life-sized room); here, the experimenter used the 3D model to demonstrate where a real toy had been hidden in the life-sized room. Finally, the child was asked to search for the real toy in the real room. To succeed, children had to transfer what they learned from one medium (e.g., the small 3D model room) to a new context (actual room). The evidence consistently revealed that transfer tasks like this are difficult for children younger than 30 months (DeLoache, 1995, 2000).

More recently, researchers have adapted this task to consider children’s ability to transfer information they learned from a video screen. The results converged well with the original findings: young children had difficulty transferring information about a hidden toy’s location from a video presentation to the real room. However, they readily transferred this information if it was presented to them in an interaction with an experimenter. This phenomenon is known as the *video deficit* (Troseth and DeLoache, 1998; Barr and Hayne, 1999; Schmitt and Anderson, 2002; Barr, 2010; see Anderson and Pempek, 2005 for a review).

Interestingly, children’s difficulty does not seem to come from screens themselves; what seems to be key is whether they have an opportunity to engage with the screen contingently.

For example, Troseth et al. (2006) adapted the task to study the effect of social interaction on 2-year-olds’ transfer ability. First, an experimenter showed the child where a toy was hidden in a room. What varied was how she showed them. Half of the children learned the toy’s location by watching a closed circuit video feed as the experimenter hid the toy (video condition); the others learned by accompanying the experimenter as she hid it in the real room (live condition). Children in the live condition successfully found the toy 77% of the time. In contrast, success in the video condition plummeted to 27%.

In a second experiment, all children learned about the hiding place from video. What varied was whether the hiding information was provided in an interactive or non-interactive fashion. In the interactive video condition, cameras were placed in both rooms and the experimenter interacted with the children throughout the hiding episode. To begin, the experimenter (with whom the child was interacting via video) played with the child for 5 minutes, establishing herself as a responsive and engaged social partner. Then, she hid the toy as children continued to watch on video. In the non-interactive control condition, children watched a 5-min recorded video of the experimenter interacting with a previous participant and then watched the experimenter hide the toy. Children in the interactive video condition successfully found the hidden toy 65% of the time; those in the non-interactive video condition succeeded at a rate of only 35%. This documents that children can indeed transfer information about the hiding location from a screen, but do so best when they are engaged with the experimenter doing the hiding.

Lauricella et al. (2010) also engaged 2½ and 3-year-old children in a transfer task, this time including an interactive computer-based condition. Children were brought into a real room and introduced to three stuffed animals who were going to “play hide-and-seek”. After becoming familiar with the room and the characters, children were brought to an adjacent room where they were randomly assigned to one of three conditions: (1) playing a “computer game” that permitted them to press a space bar to reveal the characters’ locations on a screen, (2) watching the same game unfold on the screen without interacting with it (a previously recorded video of a researcher playing the game) or (3) seeing the characters hidden by watching events taking place in the real room through a one-way mirror. As predicted, children were very successful with the one-way mirror. But they were equally successful in the interactive, bar-pressing computer game condition. Children in these conditions surpassed those in the non-interactive computer game condition. This converges with Troseth et al. (2006)’s findings, suggesting that young children learn better from contingent than non-contingent video experience.

With increasing age, children become increasingly successful at transferring what they learn from screens to other media, such as print, or real life (Aladé et al., 2016; Huber et al., 2016). Although these studies offer encouraging news about preschoolers’ ability to transfer learning from touch screens, they leave open the question of how well younger children fare.

### Word Learning Tasks

Other researchers have considered children’s ability to transfer information from screens in a different way, focusing on how successfully children learn new words from various media sources. Skype and other video chat programs are of great interest, especially since young children use them to stay in touch with distant family members. Roseberry et al. (2014) asked whether 24- to 30-month-olds could learn the meaning of new words – they focused on verbs – in three conditions: live interaction, video interaction, or *yoked* video (pre-recorded). Children were taught four novel verbs (e.g., “meeping” for a novel turning action). An experimenter performed the action while using the novel verb in complete sentences (e.g., “I’m *meeping* this toy”) in each of the three conditions. In the live interaction and video interaction conditions, children went through a warm-up period in which the experimenter addressed them by name and played with them. Children in the yoked video condition watched a previously recorded video of the experimenter as she interacted with another child via video chat. Next, children were shown clips from *Sesame Beginnings* on a split screen. On one half of the screen, the characters performed the actions matching the novel verb on which children had been trained; on the other half, they performed a non-matching action. While they watched these videos, children heard, “Where is *meeping*? Can you find *meeping*?” Children’s looking and gesturing to the two screens was recorded.

Children in the live and video interaction conditions looked at the matching action significantly longer than the non-matching action. There was no significant difference between them. Children trained in the yoked video condition, however, did not appear to learn. This lends additional support to the view that interaction is key, not whether the training occurred live or on a screen.

Additional converging evidence comes from Kirkorian et al. (2016), who measured 2-year-olds’ word learning from tablets. All children watched a tablet presentation in which an actress introduced four objects, hidden in a row of boxes. In the non-contingent condition, children watched as the experimenter continuously retrieved each object from its box and named it. In the general contingent condition, the video paused after each object was retrieved; only when children touched the screen did the story advance to the next segment (analogous to Lauricella et al., 2010’s spacebar interaction). In the specific contingent condition, children touched each individual box on the screen to see the object it contained and hear its name. Children first completed a set of training trials with four familiar animal figurines. Then, in the testing phase, they viewed four novel objects; only the last object was named (e.g., “a toma”). Next, children were asked to select the “toma” from a set of four objects placed before them. Interestingly, 30- to 36-month-olds successfully learned the word in all three conditions, but 24- to 30-month-olds were successful only in the specific contingent condition. This suggests that 24-month-olds can learn from a tablet screen, but only when they are engaged in specific contingent interaction.

In sum, young children are more successful in learning words and locations of hidden toys from screens if they are involved in *specific contingent interactions*, as compared to passively watching events unfold (Lauricella et al., 2010; Kirkorian et al., 2016).

## Story Time and Screens: The Power of Social Interaction

Research focusing on learning during story time has also identified the effects of screens and social interaction. This line of work builds on previous evidence of the advantages of *dialogic reading*, a reading style in which caregivers prompt children with questions to help engage them in the story (Whitehurst et al., 1994). Thus, current researchers tend to hold constant the child’s engagement with an adult, and to vary whether the story is presented in a book or an electronic device (Parish-Morris et al., 2013; Krcmar and Cingel, 2014; Lauricella et al., 2014).

Krcmar and Cingel (2014) recorded parent-child pairs as they read two similar stories, one presented as a traditional book and the other on an iPad screen (a still version, with no animation or interactive features). The children ranged in age from 24 to 52.5 months. Children’s comprehension from the book was significantly higher than from the iPad. Moreover, parents and children alike spontaneously offered more story-related comments and asked more story-related questions when reading the paper book. Intriguingly, parents (but not children) made more distracted (not story-related) comments in the iPad book condition. What remained unanswered was whether this advantage for books over screens at story time would change over the preschool years.

Evidence from Lauricella et al. (2014) suggests that the book advantage fades with age and experience. These researchers recorded 4-year-old children and their parents, reading both a paper book and a screen-based book. This time, the screen book had interactive features. Children’s comprehension was comparable from books and screens. There was also a hint that parents may have been slightly more engaged in the computer version, where the interactive features (e.g., clicking a character to find out more about her) were integral to the story. Apparently, then by 4 years of age, children comprehend well from books and screens, and interactive features may boost their screen learning.

Parish-Morris et al. (2013) went one step further, using ‘electronic console books’ to tease apart the contributions of screens, *per se*, and their interactive features. Electronic console (EC) books are hybrids of traditional books and touch screens: A paper book and a matching cartridge are inserted into a console, enabling sound and interactive features that can be activated by touch. Interestingly, 96% percent of the families in their sample reported having EC books at home. In the first study, Parish-Morris et al. (2013) analyzed dialogic interactions between parents and their children (either 3 or 5 years of age). Each parent-child dyad was randomly assigned to either the traditional book condition, the EC book condition or a control condition involving the EC book but with the interactive features turned off. Results revealed that parents in the EC condition provided less language related to the story and more language directed at children’s behavior (e.g., asking children to stop pressing buttons) than in the other two conditions.

In the next study, Parish-Morris et al. (2013) compared 3- and 5-year-old children’s comprehension in a new group of parent-child pairs. Dyads were assigned randomly to either a traditional book or EC book (including all the interactive features) condition. Although 5-year-olds performed at ceiling after reading books in traditional and EC formats, 3-year-olds comprehended significantly more in the traditional book than the interactive, EC book condition. What remains unclear is whether this developmental effect reflects differences in the format itself or differences in parents’ comments when reading in the two formats, and how children younger than 3 years of age fare with interactive vs traditional book formats.

## Remaining Questions

Many questions remain about how, and how well, infants and toddlers learn from touch screens. Here, we highlight three broad areas for future research.

### (1) What Apps Are Best for Very Young Children? And for What Purpose?

First, we need to understand the potential of touch screen devices to support learning in very young children, taking into account not only their abilities to engage with the screen, but also their engagement with unique features of modern touch screen devices such as localized content, cameras, and speech recognition.

Throughout history, when a new medium is introduced, it first tends to be used in the same ways as previous media. This happened with film: the very first films were moving photographs, each capturing a moment. Later, when it became possible to make longer movies, films simply portrayed live plays, with a single camera set in front of the theater stage. It took a long time before multiple cameras were used, with different angles, close-ups, etc. The same is true for television: the first TV shows were essentially radio shows in which one could see the ‘talking heads’. Also, the first news websites looked just like printed newspapers. It took some time for producers to realize how to take full advantage of the new medium. The same is likely true for tablets and smartphones: we have only scratched the surface of their capabilities.

It is currently unclear whether the perils and promise of touch screens for young children are related to something inherent about screen-learning itself or to lingering use of design choices adapted from older technologies. For example, if an electronic book is distracting, and therefore less effective than a paper book, how might distraction be ameliorated in new implementations? In their comprehensive review, Hirsh-Pasek et al. (2015) highlight the importance of social interaction, especially for the youngest children. More specifically, they argue for the value of promoting “minds-on,” active interactions that facilitate children’s ability to integrate new ideas with their existing knowledge. As technology continues to evolve and new designs become possible, ideas like these will serve as a blueprint.

After all, mobile devices with touch screens can offer experiences that weren’t possible before. Touch screens now permit a child to see herself in a story, allow parents to record stories or to describe photos in a family album, etc. More research is needed to understand how the features that are unique to touch screen technology can best be used to advance learning in young children.

### (2) When Do Infants and Very Young Children Use Touch Screens?

Second, a more careful look at the *contexts* in which parents and children use touch screens is needed. Return for a moment to the little girl and her mother on the New York subway. We all have seen caregivers using smartphones or tablets to entertain, and perhaps pacify, young children. What remains unknown is where, when, with whom and how young children use touch screens.

In a national survey, Wartella et al. (2013) provide insights into how parents use touch screen with their children. Among parents of children ranging from 0 to 8 years, 14% reported that they were “very likely” to give their child a mobile device to keep them occupied at a restaurant; 24% said they were “somewhat likely” to do so. Their reported use of mobile devices at home was lower.

If parents largely offer smartphones and tablets to their infants and young children to entertain them while they are otherwise engaged, then it would be advantageous to figure out (a) what young children actually tend to do with the devices, and (b) what kinds of apps would be most beneficial in such contexts. If parents are using the devices with their children some of the time, it is important to understand how to support, not get in the way, of parent-child interactions. For example, apps can be programmed to run differently when an adult is engaged with the child (i.e., by letting the adult, rather than the app, do the talking) than when the child is alone. As touch screen technology and the corresponding content evolves, more research is needed not only on current usage patterns, but on methodologies that track children’s use.

Smartphones and tablets can be programmed to track incredible amounts of data – provided, of course, that adequate privacy protections or consent are in place – including how long an app was used, every touch on the screen and even the location of the child during the interaction. This data would reveal how children from 0 to 3 years of age use touch screens and how (much) they learn from them.

It will also be important to identify what kinds of learning opportunities children miss out on when they are occupied with touch screens, rather than engaging with others and observing social interactions. Turkle (2012) offers considerable food for thought along these lines, articulating how our nation’s increasing engagement with digital devices come at the expense of the learning and social connections that arise naturally from real-time conversation and engagement with others. A pressing concern is how infants’ and young children’s burgeoning access to touch screens affects their ability to communicate with and relate to others.

### (3) How Does Touch Screen Adoption and Use Vary Across Cultural Communities?

Third, entirely absent from the literature thus far is a careful consideration of the role of culture. How do families from different cultural communities incorporate mobile devices into the routines of infants and very young children? Are parents hoping devices will bolster skills they don’t feel prepared to teach themselves (such as a second language, in the case of immigrant families)? What are best practices for parents and educators of children from all of our nation’s diverse communities?

We look forward to new research that will illuminate both the promise and perils of touch screens in early development.

## Author Contributions

SL and SW contributed to the critical analysis of the literature, drafting, revising, and approving the final manuscript.

## Conflict of Interest Statement

The authors declare that the research was conducted in the absence of any commercial or financial relationships that could be construed as a potential conflict of interest.
